# Cytokine-Mediated Plasticity in the Molecular Requirements for Peptide-Loading Complex Formation and Antigen Presentation

**DOI:** 10.3390/cells15201914

**Published:** 2026-10-21

**Authors:** Ritchlynn Aranha, Mohadeseh Dastpeyman, Kirti Pandey, Mohammadreza Dorvash, Rochelle Ayala, Nathan P. Croft, Anthony W. Purcell

**Affiliations:** 1 Infection and Immunity Program, Biomedicine Discovery Institute, Monash University, Melbourne, VIC 3800, Australia; ritchlynn.aranha@nyulangone.org (R.A.); mohadesedp@gmail.com (M.D.); kirti.pandey1@monash.edu (K.P.); dorvashmr@gmail.com (M.D.); rochelle.ayala@monash.edu (R.A.); nathan.croft@monash.edu (N.P.C.); 2 Department of Biochemistry and Molecular Biology, School of Biomedical Sciences, Monash University, Melbourne, VIC 3800, Australia

**Keywords:** antigen processing, antigen presentation, mass spectrometry, HLA, immunopeptidomics, cytokine signaling, immune evasion

## Abstract

**Highlights:**

**What are the main findings?**

• Interferon-γ (IFNγ) strongly amplifies the residual antigen-processing machinery in ERAP1-, TAP1- and TAP2-deficient cells, increasing surface HLA-I expression and expanding the immunopeptidome despite persistence of the underlying genetic defect.
• The extent of this rescue is component-dependent: TAP1-deficient cells show the greatest convergence towards wild-type peptide characteristics, while ERAP1- and TAP2-deficient cells retain distinct processing signatures. Peptide-loading and ER quality-control proteins also remain associated with immature HLA-I heavy chains in TAP-deficient cells.

**What are the implications of the main findings?**

• Defects in individual antigen-processing components do not necessarily constitute absolute blocks to HLA-I antigen presentation; inflammatory signalling can exploit residual and alternative processing pathways to maintain substantial peptide presentation.
• Restoration of HLA-I abundance does not equate to restoration of a wild-type immunopeptidome, indicating that both the underlying antigen-processing defect and inflammatory state determine the antigenic landscape relevant to immune surveillance, tumour immune escape and antigen-targeted therapy.

**Abstract:**

HLA class I antigen presentation depends on coordinated peptide processing, transport and loading, yet inflammatory signalling may modify the consequences of defects in this machinery. We examined wild-type HAP1 cells and isogenic ERAP1-, TAP1- and TAP2-knockout cells before and after interferon-γ (IFNγ) treatment using flow cytometry, proteomics, and immunopeptidomics. IFNγ strongly induced residual antigen-processing machinery in all HAP1-genotypes, increased HLA-I surface expression and expanded peptide recovery, including peptides derived from cancer-associated antigens. However, repertoire restoration was incomplete and defect-specific. ERAP1-deficient cells retained enrichment of longer, terminally extended peptides, whereas TAP2-deficient cells retained alterations in peptide length, processing of peptide flanking regions and source-protein location of the peptide. TAP1-deficient cells showed the greatest convergence to wards wild-type immunopeptidome features upon IFNγ stimulation. Components of the peptide-loading and ER quality-control machinery remained associated with immature HLA-I heavy chains in TAP-deficient cells and were enhanced by exposure to IFNγ. These findings demonstrate that inflammatory signalling can quantitatively and partially qualitatively compensate for defects in HLA-I antigen processing by amplifying intact pathway components and alternative peptide supply, without recreating a fully wild-type immunopeptidome.

## 1. Introduction

Human leukocyte antigen (HLA) class I molecules (HLA-I) enable the most nucleated cells to continuously “display” fragments of intracellular proteins at the cell surface for inspection by CD8^+^ T cells. In the classical HLA-I pathway, normal turnover of proteins, as well as defective ribosomal products and pathogen- or tumour-derived proteins, generate peptide precursors predominantly through the actions of the proteasome. More broadly, exogenous antigens and those that intersect various secretory pathways can also enter the class I pathway through processes such as cross-presentation and endosomal recycling of HLA complexes [1,2,3,4,5]. These peptide precursors can be further processed by cytosolic peptidases, prior to their transport into the endoplasmic reticulum (ER) by the heterodimeric transporter associated with antigen processing (incorporating TAP1 and TAP2 subunits) [6]. In the ER, the nascent HLA-I heavy chain associates with β2-microglobulin and engages a peptide-loading “assembly line” that includes calnexin, calreticulin, ERp57 and the formation of a peptide-loading complex (PLC) centred around the specialised chaperone tapasin [7,8,9,10]. The formation of the PLC links HLA-I to TAP and promotes the acquisition and editing of high-stability peptide ligands [11,12,13,14,15,16]. For many HLA allotypes, ER aminopeptidases (ERAP1 and ERAP2) trim N-terminally extended peptide precursors to optimal 8–12 mer ligands, increasing the abundance of stable HLA-I–peptide complexes that can traffic through the secretory pathway to the plasma membrane [17,18,19,20]. The result is a dynamic “immunopeptidome”, a collection of HLA class I bound peptides that reflects the cellular proteome, inflammatory state (including interferon-driven remodelling of antigen processing), and the binding specificities of an individual’s HLA allotypes [21].

This antigen-presentation system underpins immune surveillance by providing a molecular readout of intracellular health. CD8^+^ T cells scan HLA-I-bound peptides via their clonally distributed T-cell receptors and can eliminate cells presenting non-self or aberrant self-peptides arising from infection, oncogenic transformation, or cellular stress. In parallel, natural killer (NK) cells integrate signals from HLA-I and other ligands to detect “missing-self” states, providing a complementary safeguard when HLA-I expression is reduced. The breadth and sensitivity of surveillance are further extended by cross-presentation, in which professional antigen-presenting cells can acquire exogenous material and present it on HLA-I to prime CD8^+^ T-cell responses. Together these mechanisms help maintain tissue homeostasis and constrain the emergence of infected or malignant cells.

In cancer, immune surveillance and tumour evolution are often framed as immunoediting, comprising elimination, equilibrium, and escape [22]. Tumours can be eliminated when antigen presentation and effector responses are intact, but persistent immune pressure can select for cancer cell variants that evade recognition, which can commonly occur through quantitative or qualitative disruption of components of the HLA-I pathway (e.g., loss of β2-microglobulin, downregulation of TAP/tapasin/ERAP, defects in interferon signalling, or loss of HLA alleles) [23]. Such aberrations reduce tumour immunogenicity, reshape the immunopeptidome, and contribute to resistance to T-cell-based immunotherapies. Conversely, therapies that reinvigorate T-cell function (e.g., checkpoint blockade) highlight the centrality of effective antigen presentation for durable anti-tumour control, motivating efforts to understand and therapeutically modulate the antigen-processing and presentation machinery [24].

Thus, HLA-I antigen presentation is orchestrated by integrated and synergistic antigen-processing and presentation machinery (APM) that generates proteasome-derived peptides, transports them into the endoplasmic reticulum (ER), and loads high-affinity ligands onto nascent HLA-I molecules. Disruption of individual APM components can profoundly alter cell surface HLA-I abundance and reshape the presented immunopeptidome, with direct consequences for anti-viral and anti-tumour T-cell recognition and for mechanisms of immune escape in cancer. To enable controlled, component-by-component dissection of this pathway, Spaapen and colleagues generated PAKC (Panel of APM Knockout Cells): a set of isogenic human HAP1 cell lines created by CRISPR/Cas9, each lacking a single, key HLA-I pathway gene [25]. The original PAKC collection includes knockouts spanning the pathway from HLA-I expression (HLA-A/B/C and B2M) through ER peptide supply and editing (TAP1, TAP2, tapasin, ERAP1) and ER chaperoning/quality-control molecules (calnexin/CANX, calreticulin, ERp57, glucosidase IIα). These cell lines were validated at the sequence and protein levels and provide a shared genetic background in which changes in HLA-I surface expression, peptide repertoire, and T-cell recognition can be attributed to the loss of a specific APM node rather than confounded by cell-line differences. The PAKC panel has become a useful community resource for mechanistic studies of peptide loading and antigen presentation, benchmarking of immunopeptidomics workflows, and functional interrogation of immune-evasion phenotypes relevant to immunotherapy. Building on the original panel, recent work has applied integrated immunopeptidomics and proteomics across HAP1 APM perturbations to quantify how specific gene losses induce distinct, pathway-consistent shifts in peptide-length distributions, peptide ligand sequence motif usage, and antigen abundance [25,26]. Together, these isogenic knockout systems provide a tractable platform for linking APM genotype to immunopeptidome phenotype and, ultimately, to T-cell-mediated immunity.

Building on this earlier work we go on further to explore how IFNγ stimulation can compensate for some of these defects and to varying extents repair deficits in antigen presentation and reinstate wild-type-like immunopeptidomes. IFNγ drives coordinated remodelling of the HLA-I antigen-processing pathway, increasing both antigen-presentation capacity and the selectivity of peptide generation and loading onto HLA-I [27]. STAT1- and IRF1-dependent transcription promotes HLA-I and TAP expression, increasing peptide transport into the endoplasmic reticulum [28]. In parallel, induction of immunoproteasome subunits and the proteasome regulator PA28 alters proteolytic cleavage and ligand production [29,30]. IFNγ also increases components supporting HLA-I assembly and peptide loading, including β2-microglobulin and tapasin [27]. Induction of ERAP1 further modifies the peptide pool through N-terminal trimming, which can generate suitable HLA-I ligands or destroy individual peptides through overtrimming [18,19]. Together, these changes produce qualitative and quantitative shifts in the HLA-I immunopeptidome, including altered peptide abundance, length and sequence composition, rather than a uniform increase in presentation of all antigens [27].

## 2. Materials and Methods

### 2.1. Cell Lines

HAP1 is a near-haploid human cell line derived from KBM-7, a chronic myelogenous leukaemia [25]. Unlike KBM-7, HAP1 cells contain a disomy of chromosome 15, while only containing a single copy of other chromosomes [31,32]. This cell line is commonly used in genetic screening assays due to its haploid nature, and this haploidy has been used advantageously to study the phenotypic characteristics of different genes [31,33,34]. This study uses a panel of HLA class I antigen-presentation machinery HAP1-knockout cells (PAKCs), which were provided by Dr Robbert Spaapen, Sanquin Research, Amsterdam [25]. The variants used in this study include HAP1 wild-type (WT) and knockouts of TAP1, TAP2 and ERAP1. All cell lines have three common HLA-I allotypes—HLA-A*02:01, HLA-B*40:01, and HLA-C*03:04. HAP1 cell lines were grown Iscove’s Modified Dulbecco’s Medium (IMDM; Thermo Fisher Scientific, Waltham, MA, USA); supplemented with 10% foetal bovine serum (FBS; Thermo Fisher Scientific) (IMDM-10) media and incubated at 37 °C, 5% CO_2_.

T2 (174×CEM.T2) is a human T–B lymphoblastoid hybrid cell line derived from fusion of the B-lymphoblastoid line 721.174 with the T-cell line CEMR.3. T2 is a variant of the T1 hybrid that has a deletion of the MHC class II region encompassing the antigen-processing machinery [35]. It lacks functional TAP1/TAP2-mediated peptide transport, resulting in inefficient peptide loading and markedly reduced HLA class I surface expression [36]. T2 cells were cultured in RPMI 1640 medium (Thermo Fisher Scientific) supplemented with 10% FBS (RPMI-10) media and incubated at 37 °C, 5% CO_2_.

### 2.2. Quantification of Surface HLA-I Expression by Flow Cytometry

Flow cytometry was performed regularly on all cell lines to ensure uniform levels of surface HLA-I expression. Cells were washed twice with 200 µL of ice-cold PBS and incubated (30 min, 4 °C) with 100 µL of primary antibody supernatant (W6/32 (pan anti-HLA-I) [37], BB7.2 (anti-HLA A2) [38], anti-Bw6 [39] and DT9 (anti-HLA C/HLA E) [40] all produced in house from B cell hybridomas). Cells were washed twice with ice-cold PBS and incubated in 50 µL of Goat anti-mouse IgG conjugated to phycoerythrin (PE) (Southern Biotechnology Associates, UK (Cat#1032-09)) and 50 µL of LIVE/DEAD^TM^ Fixable Aqua (Thermo Fisher Scientific) for 30 min, 4 °C, in dark. Cells were washed twice with ice-cold PBS and fixed with 1% paraformaldehyde (PFA; ProSciTech, Kirwan, QLD, Australia). Cells were washed and resuspended in 300 µL of ice-cold 1X PBS, following which flow cytometry was performed using a LSRFortessa™ X-20 Flow Cytometer (BD Biosciences, San Jose, CA. USA). HLA-I surface expression was analysed using FlowJo^®^ software v10.8.1 (BD Biosciences). Geometric mean fluorescence intensity (gMFI) was used to quantitate the HLA-I levels in cell lines and data was visualised using GraphPad Prism^®^ v10.1.1 (Insight Partners, New York, NY, USA).

### 2.3. Optimisation of IFNγ Treatment—Cell Viability and Surface HLA-I Expression

HAP1 cell lines were grown in IMDM-10 for 24 h until cells reached a 60% confluence. Cell lines were then treated with varying concentrations of IFNγ (PeproTech, Cranbury, NJ, USA; Cat. No. 300-02) (20, 50, 100, 200, and 1000 International Units (IU)/mL) for 24–72 h. The IFNγ incubation time and concentration were optimised for highest cell surface HLA-I expression and cell viability as determined by flow cytometry (Figure S1).

### 2.4. Proteomics of Cell Lines

#### 2.4.1. Suspension Trapping (S-Trap) Digestion and Tryptic Peptide Isolation

HAP1 wild-type and KO cells were grown in the absence and presence of IFNγ, in triplicate, to generate a cell pellet of 2 × 10^6^ cells, following which they were flash frozen in liquid nitrogen. For extraction of the global proteome, cell pellets were lysed using 2× lysis buffer (10% SDS with 200 mM TEAB, pH 7.55), following which the lysate was sonicated using a probe sonicator to shear DNA (amplitude 10, 10 s bursts × 3). The lysate was clarified by centrifugation at 4000× *g* for 5 min, and the supernatant was transferred to a new LoBind Eppendorf tube. Protein concentration was measured using a Direct Detect^®^ Spectrometer (Merck Millipore, Burlington, MA, USA), and 100 μg of protein was reduced by 20 mM TCEP solution (ThermoFisher Scientific) and alkylated using 20 mM Iodoacetamide (IAA; Merck). Proteins were processed using S-Trap™ micro spin columns (ProtiFi, Fairport, NY, USA) according to the manufacturer’s protocol and digested with trypsin as previously described [41]. Peptides were recovered in three wash steps: 80 μL of digestion buffer followed by 80 μL of 0.2% formic acid (FA; ThermoFisher Scientific) and finally 80 µL of 0.2% FA in 50% acetonitrile (ACN; Fisher Scientific). Peptides were then frozen and vacuum concentrated. The dried samples were then reconstituted in 100 µL of 0.1% Trifluoroacetic Acid (TFA; ThermoFisher Scientific). For clean-up and concentration of tryptic peptides, 100 µL OMIX C18 tips (Agilent, Santa Clara, CA, USA) were conditioned three times in 100 µL of buffer B (0.1% FA and 50% ACN, in optima water). Tips were equilibrated with three washes of 100 µL of buffer A (0.1% FA, in optima water). Peptides were then loaded onto the tips, followed by washes with 100 µL of buffer A (×3). Peptides were eluted from the tip with 150 µL of buffer B, frozen, and vacuum concentrated and finally reconstituted in 20 µL of MS loading buffer (0.1% FA + 2% ACN). Peptide concentration was determined using a ThermoFisher Scientific NanoDrop and a total of 75 ng of peptides was spiked with 1 µL of 0.33 pmol/μL indexed retention time (iRT; GL Biochem, Shanghai, China) peptides and transferred to MS vials.

#### 2.4.2. LC-MS/MS of Tryptic Peptides

Samples were analysed using a hybrid trapped ion mobility–quadropole time of flight mass spectrometer (Bruker timsTOF Pro2, Bruker Daltonics, Bremen, Germany) coupled with a nanoElute UHPLC liquid chromatography system operated using the DIA-PASEF method. Tryptic peptides were loaded onto a Trap PepMap Neo (C18, 5 mm × 300 μm, 5 μm particle size) trap column (Thermo Fisher Scientific), eluted, and separated on an Aurora (25 cm × 75 μm internal diameter ×1.7 µm particle size) (IonOpticks, Richmond, VIC, Australia) analytical column using a linear stepwise gradient of buffer A (Optima water, 2% ACN, 0.1% FA) to buffer B (ACN, 0.1% FA). The gradient used started from 0 to 17% buffer B over 60 min, then to 25% over the next 30 min, 37% over the next 10 min followed by a rapid rise to 95% buffer B over a subsequent 10 min period with the flow rate set to 300 nL/min in dia-PASEF mode. Data-dependent acquisition was performed with the following settings: *m*/*z* range: 100–1700 mz; capillary voltage: 1600 V; target intensity of 30,000; and TIMS ramp of 0.60 to 1.60 Vs/cm^2^, with collision energy ramped linearly as a function of the mobility from 59 eV at 1/k0 = 1.6 Vs cm^−2^ to 20 eV at 1/k0 = 0.6 Vs cm^−2^. For DIA, two windows were used in each 100 ms dia-PASEF scan with 16 of these scans (32 steps) covering the doubly and triply charged peptides in the *m*/*z* range of 400–1200 *m*/*z* and singly charged peptides in the *m*/*z* range of 800–1200, using isolation windows with a width of 26 Da.

#### 2.4.3. Analysis of Proteome Data

DIA-NN software v1.8.1 [42] was used to analyse LC-DIA-MS proteomics data using the following settings—library-free search mode; precursor FDR 1%; mass accuracy at MS1 and MS2 set to 20 ppm; scan window set to 0; isotopologues turned on; MBR turned on; heuristic protein inference turned on; no shared spectra turned on; protein inference at protein names (from FASTA); neural network classifier double-pass mode; quantification strategy any LC (high precision); cross-run normalisation global; and library generation smart profiling. The database used was UniProt Human proteome (20,372 entries; version February 2022; iRT and cRAP proteins included in list). Both FASTA digest for library-free search/library generation and deep learning-based spectra RT and IM prediction were selected. Trypsin/P with 2 missed cleavages was used; Cysteine Carbamidomethylation (+57.02146) was used as the fixed modification protein N-terminal M-excision, oxidation of methionine (+15.9949), and protein N-terminus acetylation (+42.0106) were used as variable modifications. Peptide length ranged from 7 to 30; precursor charge ranged from 1 to 4; precursor *m*/*z* ranged from 300 to 1800; and fragment ion *m*/*z* ranged from 200 to 1800.

The output of the DIA-NN search (report.pg_matrix.tsv, see Table S1) was imported into DIA-Analyst for differential protein abundance analysis and visualisation (Monash Proteomic Analyst Suites, Monash University), based on the framework described by Shah et al. [43]. Data was pre-filtered to remove potential contaminant sequences and reverse sequences. Following pre-filtering, LFQ intensities were Log2-transformed, and rows were filtered to remove proteins with a high proportion of missing values (proteins that had ≥50% valid values in at least one group), following which missing values for protein intensities were imputed using Perseus-type missing value imputation. The false discovery rate (FDR) correction option selected was the Benjamini–Hochberg method. The output of DIA-Analyst was exported, the clusters were then exported in a .txt file format, and gene set enrichment analysis (GSEA) was performed using EnrichR [44,45].

#### 2.4.4. Analysis of HC10 Immunoprecipitates and Peptide-Loading Complex Inference

Five million T2, HAP wild-type or KO cells were harvested, washed with ice-cold PBS and lysed at 4 °C in a mild detergent-based lysis buffer containing 1% IGEPAL CA-630 and protease inhibitors to preserve interactions between HLA-I heavy chains and components of the ER peptide-loading and quality-control machinery as described [46]. Insoluble material was removed by centrifugation and clarified lysates were incubated with 50 µL of HC10 antibody coupled with protein A Sepharose beads (10 mg/mL) with rotation at 4 °C. Beads were washed extensively using lysis buffer containing a reduced concentration of IGEPAL CA-630 (0.1%) to remove non-specifically associated proteins while maintaining protein–protein interactions. HC10-associated proteins were subsequently reduced, alkylated and digested with trypsin as described above. Peptides were analysed by LC–DIA-MS and proteins were identified using DIANN software [42]. Relative recovery of HLA-I-associated proteins across experimental conditions was assessed using peptide–spectrum matches and spectral counts as a semi-quantitative measure of protein abundance [46].

### 2.5. HLA-I Immunopeptidomics

Immunopeptidomics was performed on HAP1 cell lines that were cultured in the presence and absence of IFNγ. Cells were grown to high density followed by regular cell pelleting until a cell pellet size of 5 × 10^8^ cells was achieved. Following harvesting, cell pellets were washed twice with ice-cold PBS, flash frozen, and stored at −80 °C until their time of use.

#### 2.5.1. Cross-Linking of Purified Antibody to Protein a Resin

A total of 1 mL of Protein A Sepharose (PAS; Cytiva, Little Chalfont, UK) was transferred into a tube containing purified antibody and rolled end-over-end for an hour at 4 °C prior to being transferred into a 10 mL Bio-Rad Poly-prep chromatography column and washed with 20 column volumes of 0.05M borate buffer at pH8.0 and the antibody was cross-linked to protein A on the column using Dimethyl pimelimidate dihydrochloride (DMP; Sigma Aldrich, Saint Louis, MO, USA). Cross-linking was quenched by the flow of 20 column volumes of ice-cold 0.2M Tris-HCl (pH 8.0) followed by 20 column volumes of 0.1M citrate buffer (pH 3.0) to strip off any unbound antibody. Cross-linked antibody–PAS was then washed with 20 mL of borate buffer and stored at 4 °C until the time of use.

#### 2.5.2. Isolation of HLA-I-Bound Peptides

HAP1 cell pellets (5 × 10^8^ cells) were lysed using a combination of mechanical and chemical techniques. Pellets were initially pulverised into a fine powder using a cryogenic mill (Mixer Mill MM 400, Retsch GmbH, Haan, Germany). Once pulverised, cells were lysed in 10 mL of ice-cold 2 × lysis buffer (1% IGEPAL CA-630 (Merck, USA), 100 mM Tris HCl (pH 8.0), and 300 mM NaCl, supplemented with cOmplete^TM^ Protease Inhibitor Cocktail (Sigma Aldrich)); the lysate was incubated for 45 min at 4 °C and rotated end-over-end. The cell lysate was then clarified by centrifugation (3166× *g*, 10 min, 4 °C), and the supernatant was transferred to a precooled ultracentrifuge tube. The clarified supernatant was then ultracentrifuged (117,524× *g*, 4 °C, 45 min; Ti70 rotor–radius: 6.57 cm) to remove cellular debris and nuclear material. Following ultracentrifugation, any lipid layer was removed from the lysate, and the clarified lysate was passed over a poly-prep column containing 1 mL of PAS to remove any non-specific binders. The flow through was then passed over the W6/32- PAS column with subsequent washing steps as described elsewhere [47]. To elute HLA–peptide complexes from PAS, 4.5 mL of 10% acetic acid was added to the column which also resulted in dissociation of the peptides from the heavy chain and β2m. The eluate mixture was further fractionated using reversed-phase high-performance liquid chromatography (RP-HPLC) to separate the peptides from the HC and β2m. RP-HPLC was performed using a 4.6 mm internal diameter × 100 mm long monolithic C18 reverse phase column (Chromolith^®^ SpeedROD, Merck) using an ÄKTAmicroTM HPLC system (Cytivia) and UNICORN^TM^ software v5.0 (Cytivia). The mobile phase buffers used in the system were buffer A (0.1% TFA (*v*/*v*) in optima water) and buffer B (80% ACN (*v*/*v*), 0.1% TFA (*v*/*v*) in optima water). The column was equilibrated at 2 mL/minute for 10 min using 2% of buffer B. Following equilibration, the eluate was injected onto the column at a flow rate of 2 mL/minute and peptides were separated using an increasing gradient of buffer B. Peptides, β2m, and HC were separated into fractions by using an increasing linear gradient of buffer B and 500 µL fractions were collected, frozen and vacuum-dried.

Following drying, samples were reconstituted in 12 µL of MS loading buffer (0.1% FA + 2% ACN) and 1 µL of 0.33 pmol/μL of iRT peptides, sonicated, and loaded onto Evotips (EvoSep, Odense, Denmark). Evotips were first washed with 20 µL of solvent B (80% ACN + 0.1% FA) and centrifuged (800× *g*, 60 s, RT), following which the tips were conditioned by soaking in 100 µL of 2-propanol. The soaked tips were then equilibrated with 20 µL of solvent A (2% ACN + 0.1% FA) and centrifuged (800× *g*, 60 s, RT). Following equilibration, the sample was loaded twice onto equilibrated Evotips and the tip was centrifuged (800× *g*, 60 s, RT). The tip was then washed with 20 µL of solvent A and centrifuged (800× *g*, 60 s, RT), followed by loading with 100 µL of solvent A and centrifugation (800× *g*, 10 s, RT).

#### 2.5.3. Analysis of HLA-I-Bound Peptides by Mass Spectrometry

For HAP1 cell lines, data-dependent acquisition (DDA) LC-MS was performed using a Bruker timsTOF Pro2 (Bruker Daltonics) coupled with an EvoSep One system (EvoSep). Peptides were loaded on a Evotip PureTM disposable trap column, and peptides were eluted and separated with a C18 Aurora Elite CSI 15 cm nanoflow UHPLC column (75 μm internal diameter, 1.7 µm particle size) (IonOpticks) kept at 50 °C using the Whisper 20 SPD method as described [48]. The mobile phases consisted of buffer A (2% ACN, 0.1% FA, in optima water) and buffer B (100% ACN, 0.1% FA).

#### 2.5.4. Peptide Identification and Immunopeptidome Analysis

Bruker timsTOF .d MS files were exported to PEAKS^®^ Online 11 software (Bioinformatics Solutions Inc.) [49] and searched against the human proteome (UniProt, version: 9 February 2022; 20,372 entries) with the contaminant database containing iRT peptides and cRAP proteins. The search was performed using the following parameters: for data refinement, the enzyme was set to none, the activation mode was set to CID, the instrument was set to timsTOF, and the acquisition was set to DDA. Once the .d files were refined, a de novo search was performed using precursor and fragment mass error tolerances set to 15 ppm and 0.05 Da respectively, with the enzyme set to none, two variable PTMs (M [+15.99] and NQ [+0.98]), and a maximum of three PTMs for each peptide. Following the de novo search, PEAKS DB (in-depth de novo assisted search) was performed. The following settings were used: mass correction and associate features with chimaera spectra were turned on, precursor mass error tolerance was set to 15 ppm, fragment mass error tolerance was set to 0.05 Da, digest mode was set to unspecific; there were two variable PTMs (M [+15.99] and NQ [+0.98]), there was a maximum of three PTMs for each peptide, with the peptide length between 7 and 30 amino acids long, and deep learning boost was turned on.

The most probable HLA restriction of each identified peptide was inferred using NetMHCpan-4.1 [50] by assigning it to the endogenous HAP1 HLA class I allotype with the lowest eluted ligand percentile rank (%Rank_EL). Peptides were classified as strong predicted binders (%Rank_EL < 0.5) or weak predicted binders (0.5 ≤ %Rank_EL < 2). Peptides with %Rank_EL ≥ 2 for all three allotypes were left unassigned.

### 2.6. Statistical Analysis

Unless otherwise stated, quantitative data are presented as mean ± standard deviation (SD), and n denotes independent biological replicates generated from separately cultured cell populations. Statistical analyses were performed using GraphPad Prism v10.1.1 (GraphPad Software, LLC, Boston, MA, USA) or within DIA-Analyst for proteomic data. All statistical tests were two-sided, and *p* < 0.05 was considered statistically significant unless correction for multiple testing was applied, in which case an adjusted *p*-value < 0.05 was required.

For flow-cytometric analysis of cell-surface HLA-I expression, geometric mean fluorescence intensity (gMFI) values were obtained from three independent biological replicates (n = 3) for each genotype and treatment condition. Effects of genotype and IFNγ treatment were assessed using two-way analysis of variance (ANOVA), followed by Šídák’s multiple-comparisons test for prespecified comparisons between genotypes within each treatment condition and between untreated and IFNγ-treated cells within each genotype. This analysis was also used to assess differences between TAP1KO and TAP2KO cells.

For global proteomic analysis, three independent biological replicates (n = 3) were analysed for each genotype under untreated and IFNγ-treated conditions. Differential protein abundance was assessed in DIA-Analyst using two-sided Student’s *t*-tests comparing untreated and IFNγ-treated samples within each genotype. Multiple-testing correction was performed using the Benjamini–Hochberg procedure to control the false-discovery rate. Proteins were considered significantly differentially abundant at an adjusted *p*-value < 0.05 and an absolute log2 fold change ≥ 1.

For immunopeptidomic analyses, three independent biological preparations (n = 3) were analysed for each genotype and treatment condition. Total peptide recovery, peptide-length distributions, repertoire overlap, predicted HLA-allotype assignment, terminal-extension frequencies, source-protein positional distributions and numbers of peptides derived from leukaemia-associated antigens were principally used as descriptive measures of repertoire composition and are reported as individual values or mean ± SD, as appropriate. Set-intersection analyses and UpSet plots represent presence/absence relationships between identified peptide repertoires and were not subjected to inferential statistical testing. Similarly, predicted HLA-binding assignments represent categorical annotations and were analysed descriptively.

For comparisons of peptide hydrophobicity, GRAVY-score distributions were compared between knockout and HAP1WT immunopeptidomes within the corresponding treatment and peptide-length groups using two-sided Mann–Whitney U tests. Where multiple peptide-length or genotype comparisons were performed, *p*-values were corrected using the Benjamini–Hochberg procedure, with an adjusted *p*-value < 0.05 considered significant.

For HC10 affinity-purification mass spectrometry, spectral counts from two independent biological samples (n = 2) were used as a semi-quantitative measure of recovery of HLA-I-associated proteins. Values are reported as the mean spectral count and visualised as log2(mean spectral count + 1). Given the limited number of independent samples, these data were interpreted descriptively and no inferential statistical testing was applied.

## 3. Results

### 3.1. Knockout of APM Components Differentially Impacts Surface HLA-I Expression

**Figure 1:**
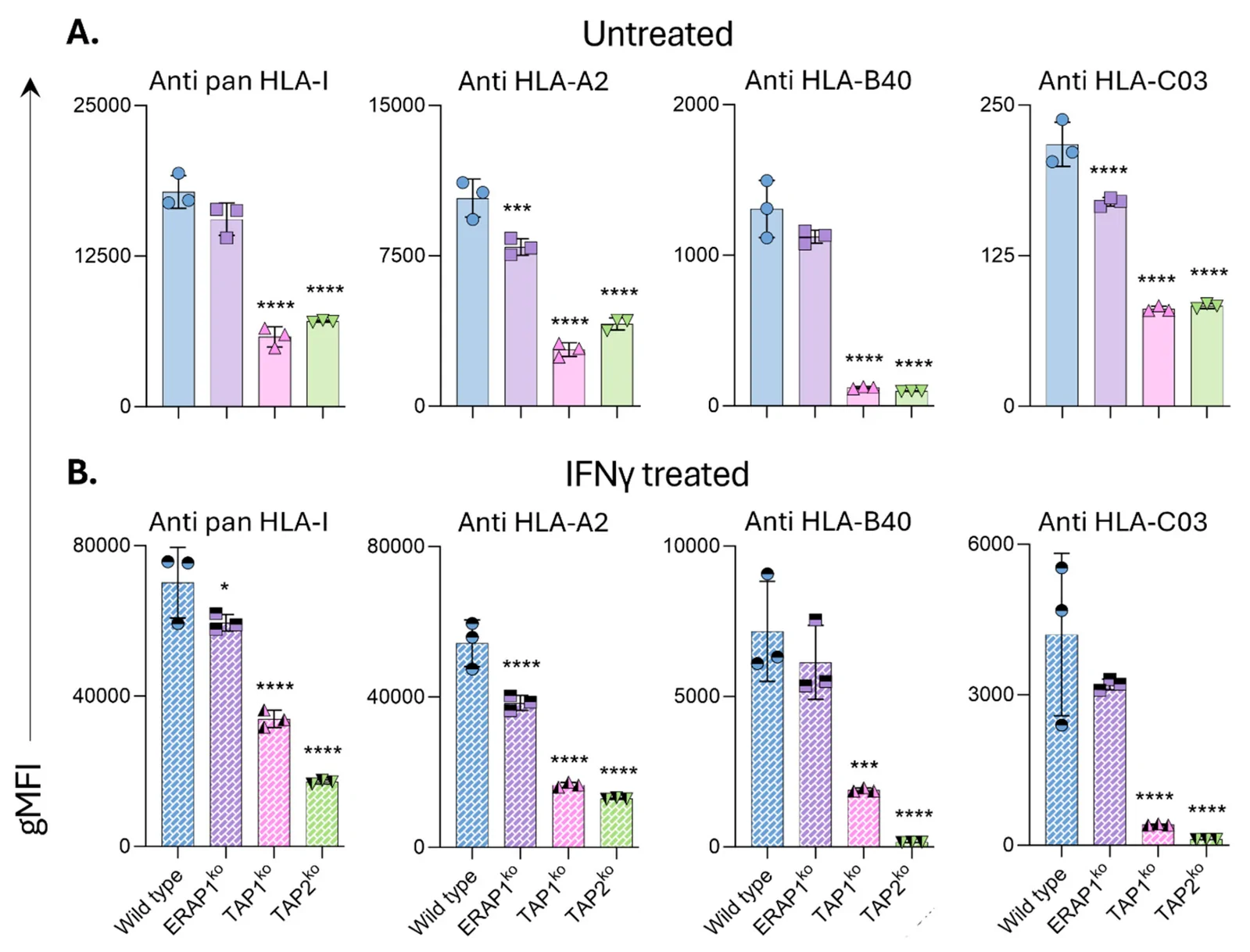
**Knockout of APM components significantly impacts surface HLA-I expression.** To examine the effect of IFNγ on cell surface HLA-I expression in HAP1 cell lines with KO of various components of the APM, flow cytometry was performed using four primary antibodies, using anti-pan-HLA-I (W6/32), anti-HLA-A2 (BB7.2), anti-HLA-B40 (Bw6), and anti-HLA-C03 (DT9) antibodies respectively. Of note, DT9 also displays cross-reactivity with HLA-E so this value represents a composite staining level. The graphs represent gMFI HLA-I expression of HAP1^WT^, HAP1-ERAP1^KO^, HAP1-TAP1^KO^, and HAP1-TAP2^KO^ in (**A**) untreated cells and (**B**) IFNγ-treated cells. All bar graphs represent (mean ± SD), n = 3. * *p* < 0.05, *** *p* < 0.001, and **** *p* < 0.0001.

Cell surface HLA-I expression is dependent on the optimal function of various APM components. Previous studies however have shown that this expression is modulated following the knockout of APM components [18,25]. To determine the impact of knocking out ERAP1, TAP1 or TAP2 on the presentation of the HLA-I alleles, selected PAKC KO cell lines (which express three HLA-I allotypes (HLA-A*02:01, HLA-B*40:01, and HLA-C*03:04)) were treated in the absence or presence of 20 IU/mL IFNγ for 24 h. Specific HLA-allotype cell surface expression was quantified by flow cytometry using four primary antibodies: W6/32 (pan HLA-I), BB7.2 (HLA-A2-specific), anti-Bw6 (HLA-B40-specific in this context) and DT9 (HLA-C-specific). For all cell lines, flow cytometry revealed that, as expected, IFNγ treatment led to a significant increase in HLA-I cell surface expression. HAP1^WT^ cells expressed high levels of surface HLA-I in both untreated and IFNγ-treated conditions (Figure 1A,B and Figure S1), whilst APM knockout modulated cell surface HLA-I expression in an allele-dependent manner. ERAP1^KO^ led to a significant decrease in HLA-A2 surface expression in both untreated and IFNγ-treated cells (Figure 1A,B). However, no significant decrease was observed in HLA-B40 or HLA-C3 expression (Figure 1A,B). Knockout of TAP1 and TAP2 on the other hand led to a significant decrease in surface expression of all three allotypes that was partially restored upon IFN-g treatment (Figure 1A,B).

### 3.2. APM Components Dominate the IFNγ-Induced Proteomic Response Despite Loss of ERAP1, TAP1, or TAP2

**Figure 2:**
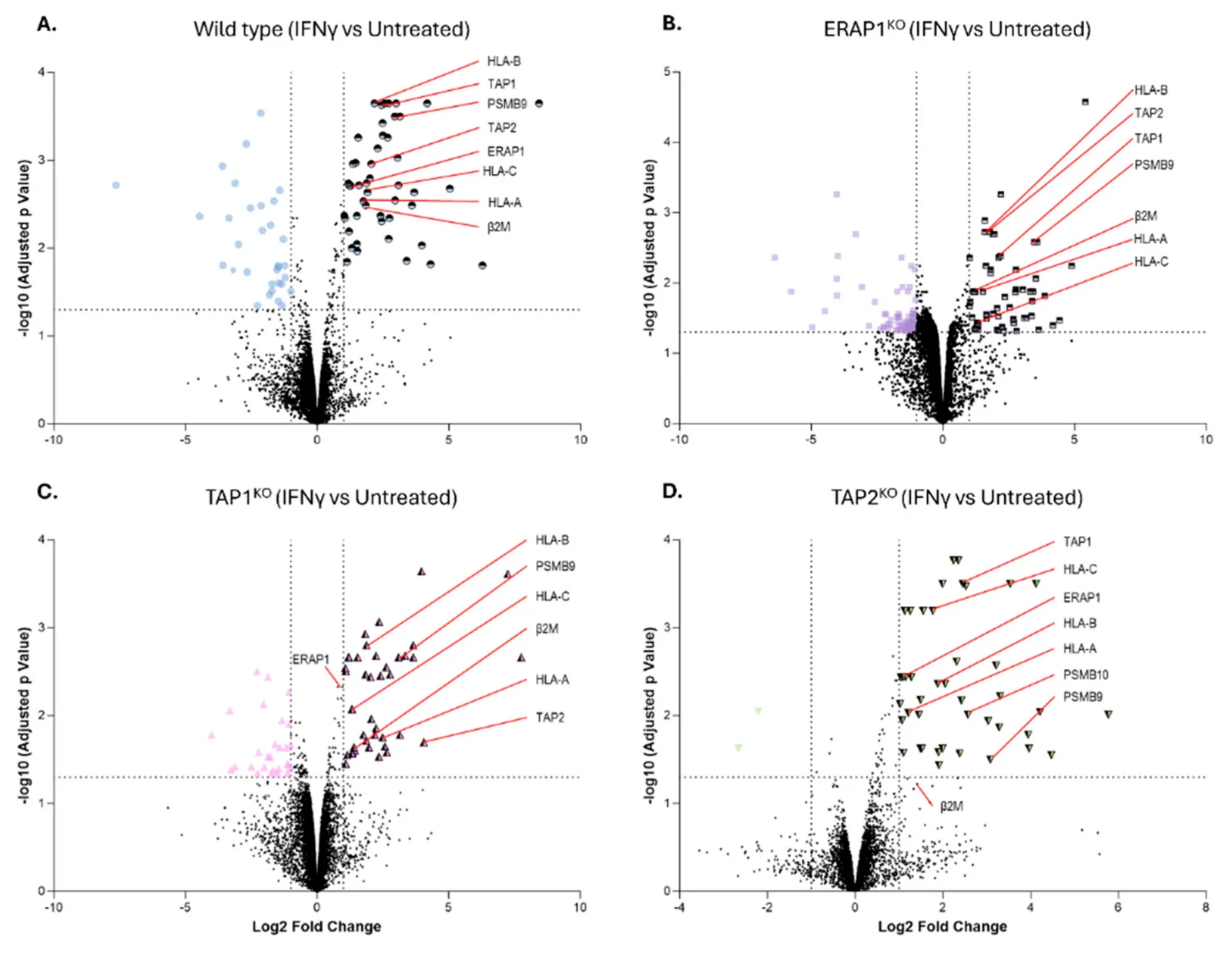
**IFNγ treatment significantly modulates the expression of APM proteins**. Volcano plots of protein expression showing significantly up/downregulated proteins in the absence and presence of IFNγ in (**A**) HAP1-WT cells, (**B**) -ERAP1^KO^ cells, (**C**) -TAP1^KO^ cells, and (**D**) -TAP2^KO^ cells. Significance was determined following a two-sided Student’s *t*-test (n = 3; adjusted −log10 *p*-value cutoff = 0.05; log2 fold change = 1).

To determine whether the partial restoration of HLA-I surface expression reflected broader remodelling of the antigen-presentation pathway, we next compared the proteomes of untreated and IFNγ-treated cells within each genotype. In HAP1^WT^ cells, IFNγ induced a prominent, coordinated increase in multiple APM proteins, including HLA-A, HLA-B, HLA-C, β2-microglobulin (B2M), TAP1, TAP2, ERAP1, and the immunoproteasome component PSMB9 (Figure 2A). These proteins were among the most strongly induced species in the IFNγ-responsive proteome, consistent with broad activation of the HLA-I antigen-presentation axis (Table S1). A similarly coordinated response was retained in each knockout line despite the absence of the targeted APM component, with the knockout confirmed by an absence of proteomic data. ERAP1^KO^ cells strongly increased HLA-I heavy chains, B2M, TAP1, TAP2, and PSMB9 (Figure 2B). TAP1^KO^ cells upregulated HLA-A, HLA-B, HLA-C, B2M, TAP2, ERAP1, and PSMB9 (Figure 2C), whereas TAP2^KO^ cells upregulated TAP1, ERAP1, HLA-I heavy chains, and the immunoproteasome subunits PSMB9 and PSMB10 (Figure 2D). The deleted protein was not detected, but the remaining pathway components retained strong IFNγ responsiveness. These data indicate that the IFNγ signalling response remains functionally intact in the APM knockout cells and can substantially amplify the residual antigen-processing capacity, providing a molecular basis for the partial restoration of HLA-I expression observed by flow cytometry.

### 3.3. IFNγ Expands and Partially Repairs APM-Deficient Immunopeptidomes

**Figure 3:**
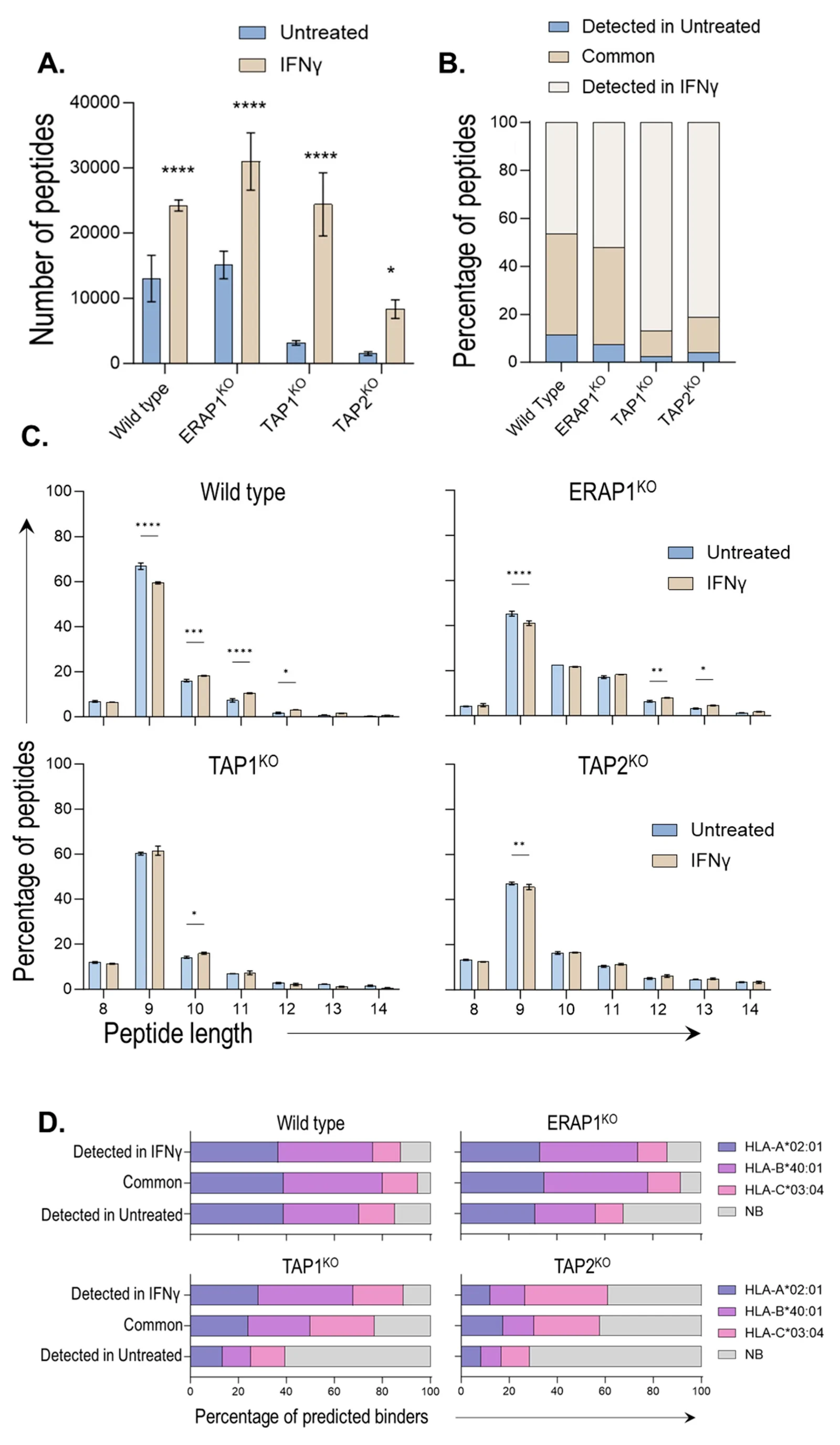
**IFNγ expands and remodels the immunopeptidome of HAP1 cells**. HAP1 cell lines were treated without or with IFNγ (20 IU/mL, 24 h), followed by W6/32 immunoaffinity purification, RP-HPLC, LC-MS/MS, and peptide identification. (**A**) Total detected peptides. (**B**) Percentage of peptides detected only in untreated cells, common to both conditions, or detected only after IFNγ treatment. (**C**) Distribution of 8- to 14-mer HLA-bound peptides. Bars show mean ± SD, n = 3. * *p* < 0.05, ** *p* < 0.01, *** *p* < 0.001, and **** *p* < 0.0001. (**D**) Peptides were grouped as detected only in untreated cells, common to untreated and IFNγ-treated cells, or detected only after IFNγ treatment. Stacked bars show the proportions of the peptides identified that are predicted to bind HLA-A*02:01, HLA-B*40:01, or HLA-C*03:04; NB indicates peptides not predicted to bind any of the three HLA-I allotypes.

We next asked whether IFNγ induction of the remaining APM components translated into increased peptide antigen presentation. IFNγ markedly increased the number of HLA-I-bound peptides recovered from every HAP1 genotype (Figure 3A, Table S2). The increase was evident in HAP1^WT^ and ERAP1^KO^ cells and was especially pronounced in the TAP-deficient lines, which had very low peptide yields under basal conditions. Following IFNγ treatment, the TAP1^KO^ immunopeptidome expanded to a depth approaching that of HAP1^WT^ cells, whereas TAP2^KO^ cells showed a substantial but incomplete recovery. ERAP1^KO^ cells generated the largest peptide repertoire after IFNγ treatment, consistent with preserved peptide transport combined with altered ER trimming.

IFNγ altered not only the depth but also the composition of the peptide repertoire. Treatment-overlap analysis further showed that the expansion was driven largely by peptides detected only after IFNγ exposure, particularly in TAP1^KO^ and TAP2^KO^ cells (Figure 3B). The peptide-length distribution remained dominated by canonical 9-mers, although IFNγ modestly reduced the relative 9-mer contribution (Figure 3C). ERAP1^KO^ cells showed the expected broader length distribution, with fewer 9-mers and proportionally more 10- to 13-mers.

We then examined whether the expanded repertoires retained sequence features compatible with the three endogenous HLA-I allotypes. Across the complete immunopeptidomes, IFNγ reduced the proportion of peptides not predicted to bind HLA-A*02:01, HLA-B*40:01, or HLA-C*03:04 in HAP1^WT^ and, more prominently, in the TAP-deficient cell lines (Figure 3D). The change was accompanied by increased assignment to HLA-B*40:01 in TAP1^KO^ cells and to HLA-C*03:04 in TAP2^KO^ cells based on NETMHC predictions. Separation of peptides according to whether they were untreated-specific, shared, or IFNγ-specific revealed that the newly induced peptide pools in TAP1^KO^ and TAP2^KO^ cells still contained a substantial fraction of peptides not predicted to bind any of the three HLA allotypes (Figure 3D). Therefore, IFNγ increased recovery of canonical HLA-associated ligands in TAP-deficient cells while simultaneously exposing a sizeable non-canonical or poorly predicted component of the rescued peptide repertoire.

**Figure 4:**
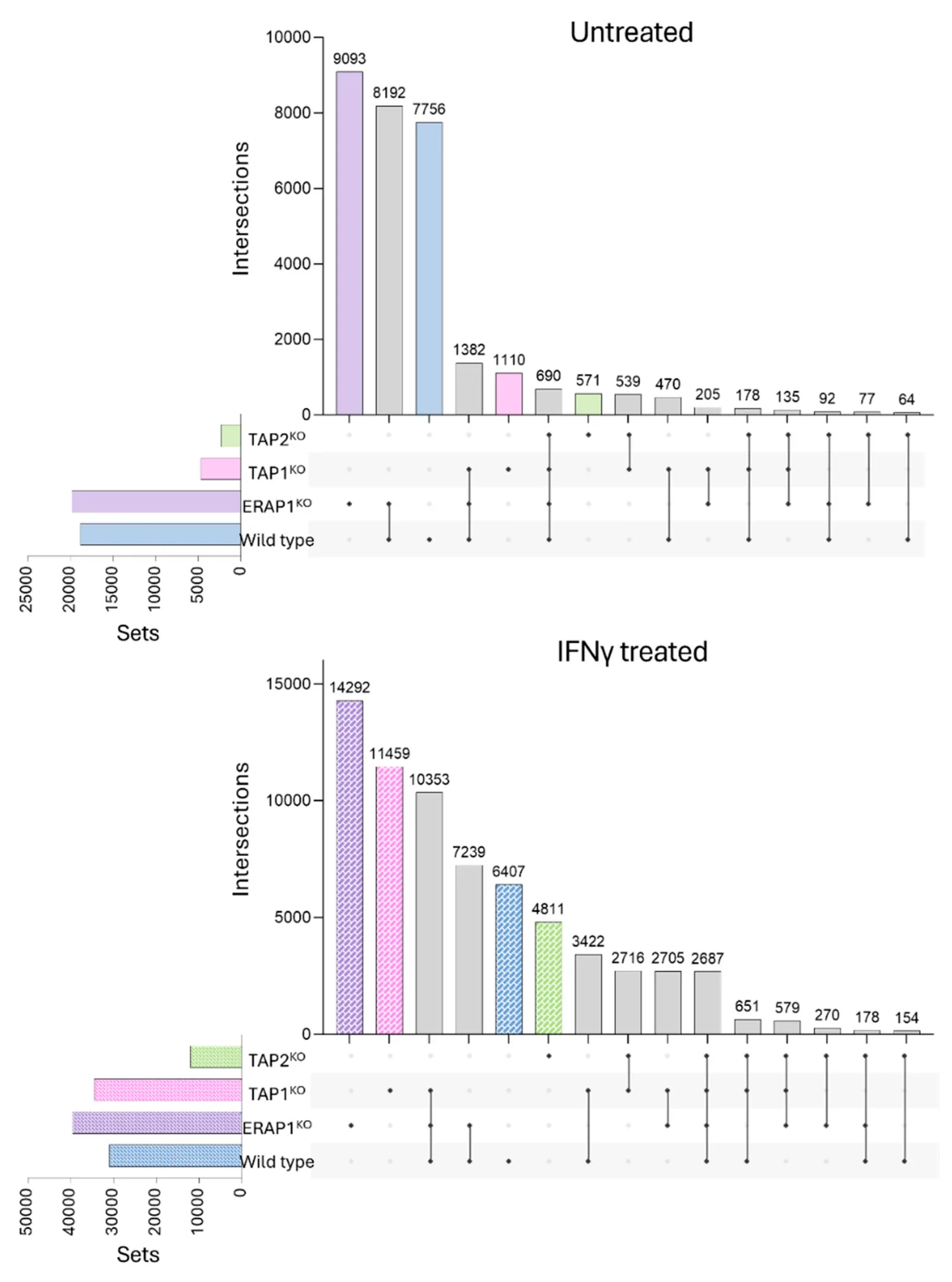
**APM knockout cells retain distinct peptide repertoires after IFNγ treatment**. UpSet plots show peptide-set sizes and intersections among HAP1^WT^, ERAP1^KO^, TAP1^KO^, and TAP2^KO^ cells under untreated and IFNγ-treated conditions. Rows indicate the cell-line peptide sets and columns indicate their intersections.

The effect of IFNγ on repertoire identity was next assessed across genotypes using intersection analysis (Figure 4). Untreated cells contained large genotype-restricted peptide populations, demonstrating that loss of ERAP1, TAP1, or TAP2 produces qualitatively distinct HLA-I repertoires rather than simply reducing repertoire size. IFNγ increased the total number of peptides in all four cell lines and generated additional shared intersections, consistent with partial convergence on an interferon-responsive antigen-presentation programme. Nevertheless, large genotype-specific peptide sets remained after treatment. Accordingly, restoration of peptide yield and surface HLA-I expression did not equate to restoration of a wild-type immunopeptidome; the identity of the deleted APM component continued to strongly determine which peptides were presented.

### 3.4. ERAP1 and TAP Deficiency Alter Peptide Terminal Processing, Hydrophobicity, and Source-Protein Position

**Figure 5:**
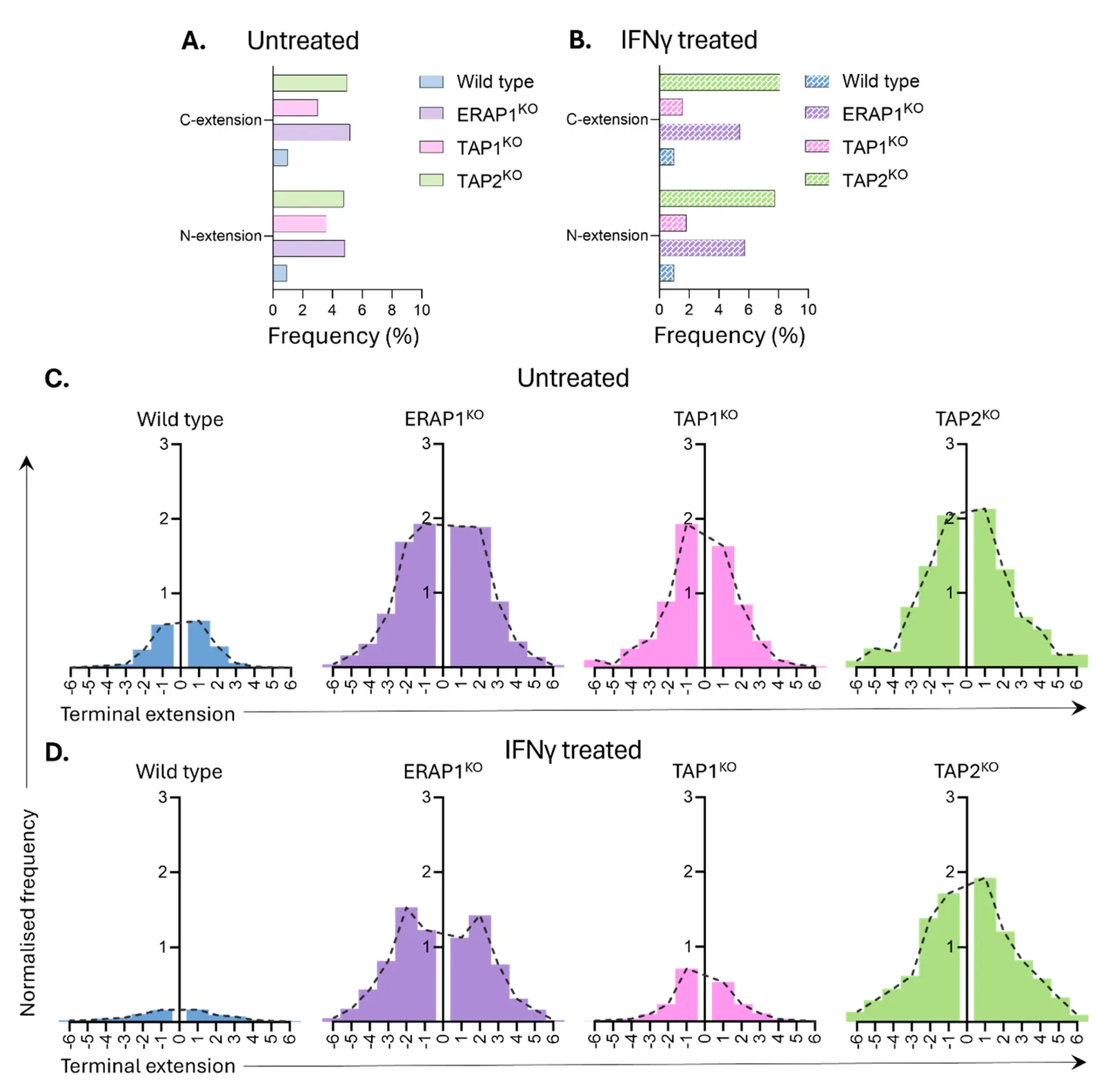
**ERAP1^KO^ and TAP2^KO^ cells preferentially present N- and C-terminally extended peptides**. Peptide extensions were assessed relative to an eight-amino-acid core. (**A**,**B**) Frequencies of N- and C-terminal extensions in untreated and IFNγ-treated cells. (**C**,**D**) Normalised distributions of terminal extensions from −6 to −1 residues at the N terminus and +1 to +6 residues at the C terminus.

To further define the processing features of the knockout-specific ligands, we examined terminal extensions relative to an eight-residue core. In untreated cells, ERAP1^KO^ and TAP2^KO^ immunopeptidomes showed the strongest enrichment of both N- and C-terminally extended peptides, whereas HAP1^WT^ contained comparatively few extended species (Figure 5A,C). TAP1^KO^ cells displayed an intermediate extension phenotype. Following IFNγ treatment, terminal-extension frequencies in TAP1^KO^ cells were reduced toward HAP1^WT^ levels, consistent with the broader evidence for partial immunopeptidome repair in this line. In contrast, extended peptides remained prominent in ERAP1^KO^ cells and were especially evident in TAP2^KO^ cells (Figure 5B,D). Thus, increased peptide presentation after IFNγ does not bypass the characteristic processing defects associated with loss of ERAP1 or TAP2. An accumulation of N-terminally extended ligands is the processing feature most directly attributable to loss of ERAP1. However, C-terminally extended ligands are a documented consequence of ERAP1 depletion, with the strongest precedent in human HLA-B27 and HLA-B*51:01 studies [51]. Such C-terminal and overall length changes likely reflect upstream proteolysis, altered peptide supply, HLA-allotype selection, and peptide editing. TAP1 and TAP2 do not trim peptides; thus, the effects observed by TAP subunit loss on peptide length are indirect, arising from altered transport selectivity and access to peptide pools that are subsequently edited/truncated in the ER and loaded onto HLA-I molecules.

APM disruption also changed the physicochemical properties of the presented ligands. Across several peptide-length classes, peptides unique to the knockout lines showed shifts toward higher grand average of hydropathy (GRAVY) scores relative to HAP1^WT^, with the most prominent differences observed among 8- to 11-mers (Figure S2). These differences persisted after IFNγ treatment and remained significant for multiple length classes (Figure 5B), indicating that cytokine-driven increases in HLA-I expression and peptide yield do not eliminate the altered hydrophobicity profile created by APM disruption. The data therefore support a role for the APM in shaping not only peptide abundance and length but also the physicochemical space sampled by the HLA-I immunopeptidome.

**Figure 6:**
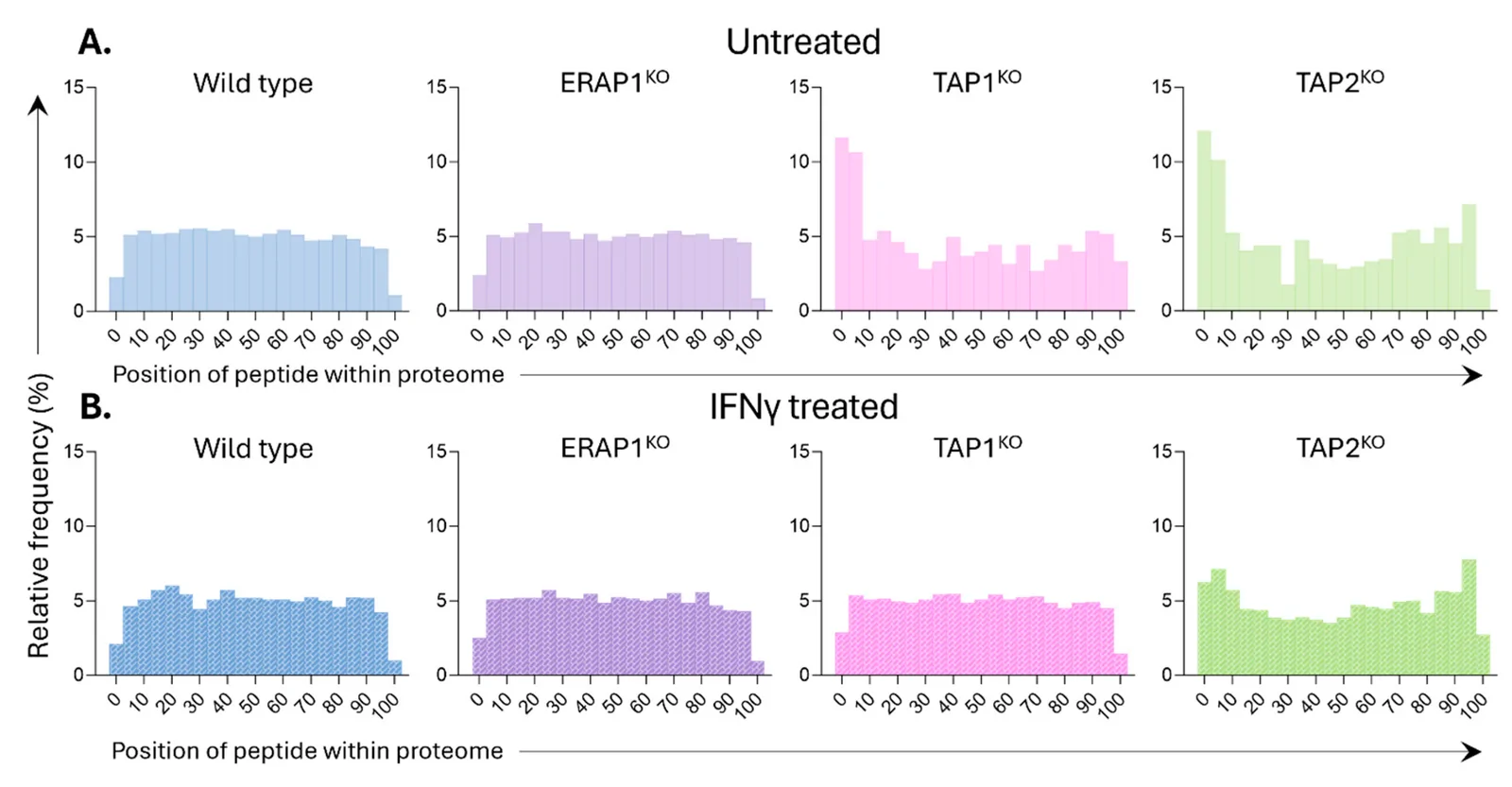
**TAP1^KO^ and TAP2^KO^ cells show altered source-protein positional bias.** The position of each presented peptide within its parental protein was normalised and distributed across 20 equal bins (vigintiles). Histograms show relative peptide frequency across the source-protein sequence in (**A**) untreated and (**B**) IFNγ-treated HAP1WT, ERAP1^KO^, TAP1^KO^, and TAP2^KO^ cells.

Finally, we mapped presented peptides to their relative position within the source protein. Peptides from HAP1^WT^ and ERAP1^KO^ cells were distributed broadly across parental proteins, with relatively little positional bias (Figure 6A). By contrast, untreated TAP1^KO^ and TAP2^KO^ cells were strongly enriched for peptides derived from the N-terminal portion of source proteins (peptides located wholly within annotated signal sequences constituted 1.1% of the untreated WT repertoire, compared with 9.1% in TAP1^KO^ and 14.5% in TAP2^KO^). IFNγ largely flattened this positional bias in TAP1^KO^ cells, producing a distribution more similar to HAP1^WT^, whereas TAP2^KO^ cells retained a pronounced enrichment at the N terminus and additional terminal skewing (Figure 6B). This differential response again distinguishes TAP1 and TAP2 loss and suggests that IFNγ can restore access to a broader source-protein landscape in TAP1-deficient cells more efficiently than in TAP2-deficient cells.

### 3.5. Peptide-Loading Complex Components Remain Associated with Immature HLA-I Heavy Chains in TAP-Deficient Cells and Are Enhanced by IFNγ

**Figure 7:**
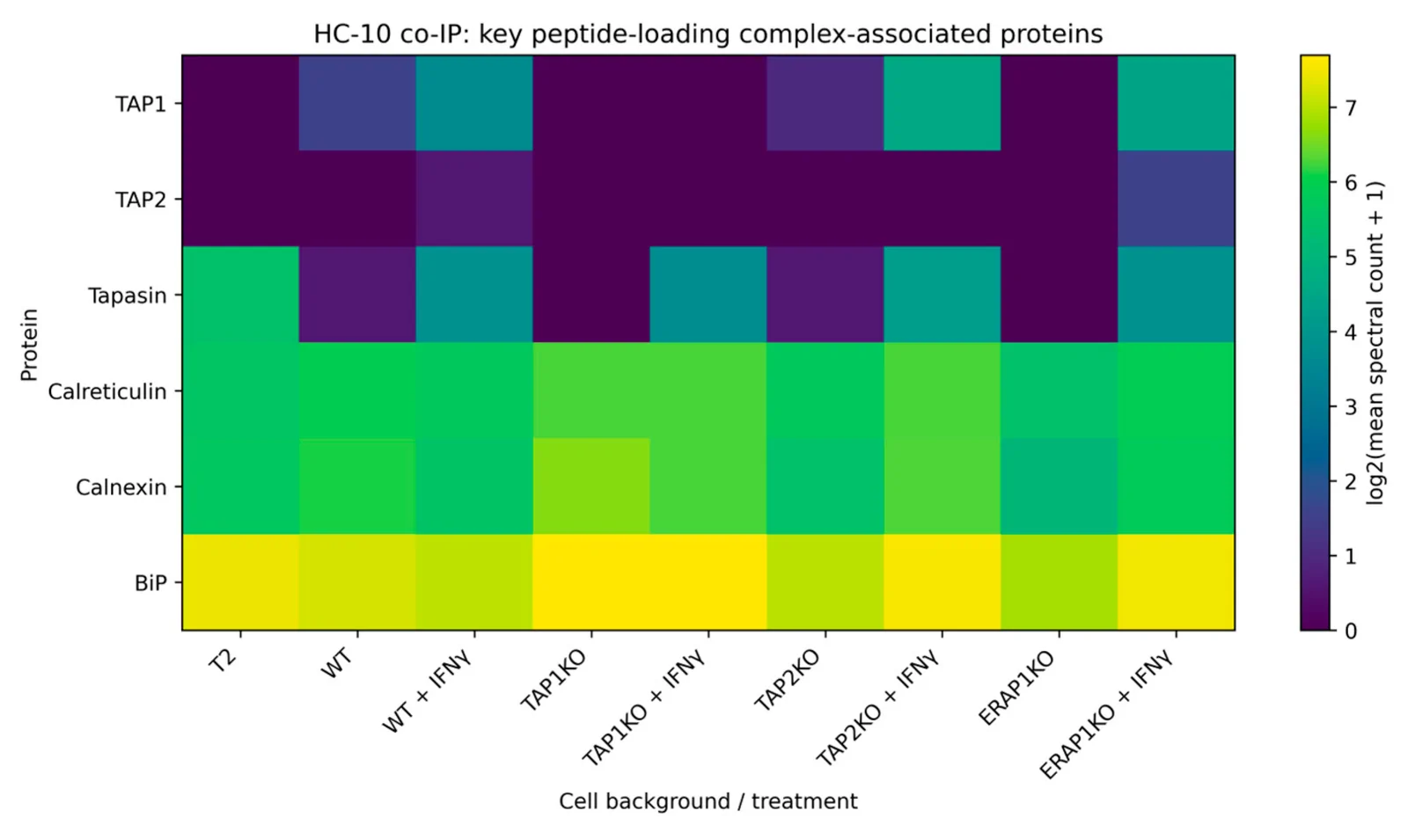
Peptide-loading complex components remain associated with HC-10-reactive HLA class I heavy chains in cells deficient in antigen-processing machinery.

We next assessed which components of the peptide-loading complex (PLC) remained associated with HLA-I heavy chains under conditions of disrupted antigen processing. For this, we used the HC10 antibody, which preferentially recognises peptide-receptive or incompletely assembled MHC-I heavy chains, to immunoprecipitate HLA-I and examine associated PLC components. Notably, HC10 immunoprecipitation continued to recover components of the HLA-I peptide-loading machinery following loss of an individual TAP subunit (Figure 7). HC-10 immunoprecipitation was performed from HAP1^WT^, TAP1^KO^, -TAP2^KO^, and -ERAP1^KO^ cells cultured in the absence or presence of IFNγ, together with TAP1/TAP2-deficient T2 cells. Co-immunoprecipitated proteins were identified by LC–MS/MS and spectral counts were used as a semi-quantitative measure of protein recovery. The heatmap shows key peptide-loading complex (PLC) and ER-associated proteins, including TAP1, TAP2, tapasin, calreticulin, calnexin and immunoglobulin-binding protein (BiP). Values represent the mean spectral count from two independent samples and are visualised as log2(mean spectral count + 1) (Supplemental Table S3). Retention of tapasin, calreticulin, calnexin and BiP in HC-10 immunoprecipitates from TAP-deficient cells indicates that immature HLA-I heavy chains remain associated with components of the ER peptide-loading and quality-control machinery despite disruption of the canonical TAP transporter. IFNγ treatment increased the recovery of several PLC-associated proteins, most notably tapasin, in the knockout backgrounds.

HC10 preferentially recognises immature or peptide-receptive HLA-I heavy chains; thus, these findings suggest that disruption of the canonical TAP1–TAP2 transporter does not abolish engagement of nascent HLA-I with ER-resident loading and quality-control factors. Consistent with this interpretation, the HLA-I–tapasin association has previously been demonstrated in TAP2-deficient T2 cells expressing TAP1 alone [52], and partially folded HC10-reactive HLA-I species can associate with tapasin–ERp57 complexes [53,54]. Thus, residual HLA-I–PLC interactions may persist in TAP-deficient cells even though canonical TAP-dependent peptide transport is lost.

### 3.6. IFNγ Broadens Presentation of Leukaemia-Associated Antigen-Derived Peptides in APM-Deficient Cells

**Figure 8:**
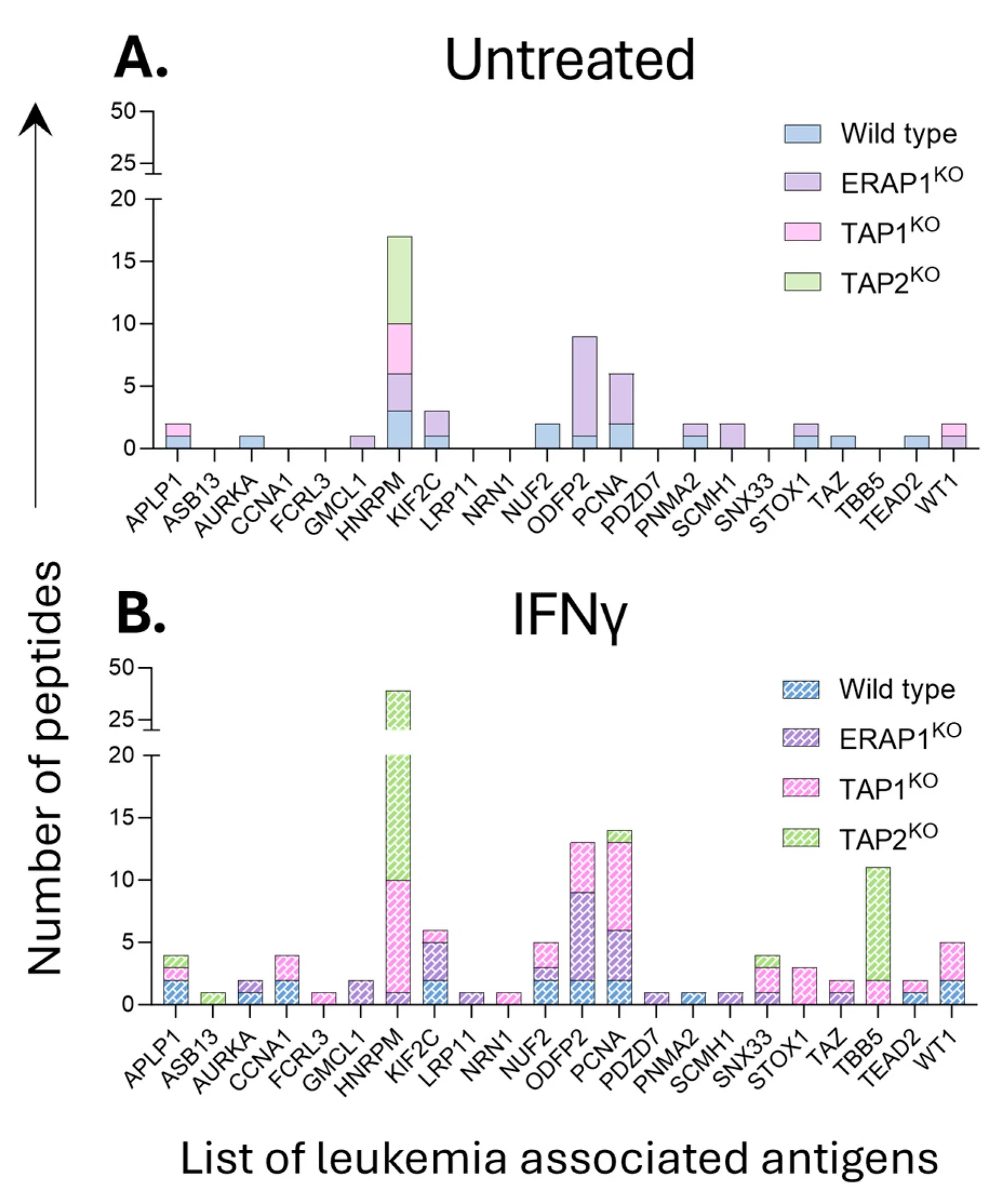
**IFNγ increases presentation of leukaemia-associated antigen-derived peptides in APM-deficient cells.** Total numbers of peptides derived from the indicated leukaemia-associated antigens are shown for (**A**) untreated and (**B**) IFNγ-treated HAP1^WT^, ERAP1^KO^, TAP1^KO^, and TAP2^KO^ cells.

To assess whether the observed changes extended to potentially disease-relevant antigens, we interrogated the immunopeptidomes for peptides derived from a curated set of leukaemia-associated antigens (LAAs). In untreated cells, LAA-derived peptide presentation was sparse and strongly genotype-dependent, with loss of TAP1 or TAP2 altering both the number and source of detectable LAA ligands (Figure 8A). IFNγ substantially increased the breadth and number of LAA-derived peptides across the panel (Figure 8B). Importantly, peptides from multiple LAAs that were absent or poorly represented under basal conditions became detectable in the TAP-deficient cells following treatment. The rescue was not uniform across antigens or knockout genotypes, indicating that cytokine stimulation expands clinically relevant antigen presentation without erasing the underlying APM-dependent selectivity.

Collectively, these data show that IFNγ drives a powerful compensatory antigen-presentation program in HAP1 cells. Upregulation of the remaining APM increases HLA-I surface expression, expands peptide yield, increases presentation of predicted HLA binders, and broadens LAA-derived antigen display. However, the extent of repair is component-specific. TAP1^KO^ cells show substantial recovery toward wild-type-like peptide length and source-position characteristics, whereas TAP2^KO^ and ERAP1^KO^ cells retain pronounced processing signatures. Thus, IFNγ produces a quantitatively expanded but not fully wild-type-equivalent immunopeptidome in cells with defined defects in HLA-I antigen processing.

## 4. Discussion

The present study uses the isogenic HAP1 Panel of Antigen-Processing Machinery Knockout Cells to examine how an inflammatory stimulus interacts with defined defects in HLA class I antigen processing. The principal finding is that IFNγ can compensate substantially for loss of individual antigen-processing components at the level of HLA-I abundance and peptide yield, but this compensation does not recreate a fully wild-type antigen-processing state. Rather, the extent and character of “repair” are strongly component-dependent. TAP1-deficient cells showed the greatest convergence toward the wild-type immunopeptidome after IFNγ treatment, whereas TAP2- and ERAP1-deficient cells retained prominent signatures of altered antigen processing. These data extend the original description of the PAKC resource and the recent systematic analysis of its basal immunopeptidomes [25,26] by demonstrating that inflammatory signalling acts on, but does not erase, the molecular consequences of individual APM lesions.

The proteomic and flow-cytometric data provide a mechanistic framework for this partial compensation. IFNγ induced a coordinated increase in HLA-A, HLA-B, HLA-C, B2M and multiple peptide-processing components, including TAP1/TAP2 where genetically intact, ERAP1, and immunoproteasome subunits such as PSMB9 and PSMB10. This is consistent with the well established role of IFNγ in transcriptionally coordinating the HLA-I pathway and shifting proteasomal processing toward an immunoproteasome-dominated state; indeed, ERAP1 was originally characterised as an IFNγ-inducible ER aminopeptidase [19]. Importantly, the targeted protein remained absent in each knockout, indicating that the response represents amplification of the residual pathway rather than reversal of the genetic lesion. This distinction is particularly clear in the surface-expression data. ERAP1 loss preferentially reduced HLA-A*02:01 while comparatively preserving HLA-B*40:01 and HLA-C*03:04, whereas loss of either TAP subunit broadly reduced all three allotypes. Such allele-selective effects are consistent with a previous analysis of the PAKC panel, which showed that perturbation of individual APM components can produce HLA-allotype-specific changes rather than uniform effects across the entire class I repertoire [26].

IFNγ treatment also produced extensive qualitative remodelling of the immunopeptidome. In each HAP1 genotype investigated, cytokine exposure increased the number and diversity of recovered HLA-I ligands, with a large proportion of the expanded repertoire comprising peptides detected only after the IFNγ treatment. Thus, IFNγ did not simply increase the abundance of peptides already presented under basal conditions. This behaviour is consistent with immunopeptidomic studies in tumour cells showing that IFNγ modifies antigen presentation through several superimposed mechanisms, including changes in source-protein abundance, induction of the immunoproteasome, increased HLA-I expression and allotype-specific changes in peptide loading [27,55]. IFNγ-driven inflammation can also generate qualitatively distinct or aberrant peptide products that are poorly represented in untreated cells [56,57,58]. The current data extend these observations to cells carrying defined APM defects: even when peptide supply or ER trimming is genetically compromised, IFNγ can markedly broaden the detectable peptide repertoire. However, the continued presence of large genotype-specific peptide sets and the substantial fraction of IFNγ-induced peptides not assigned by prediction to HLA-A*02:01, HLA-B*40:01 or HLA-C*03:04 emphasise that increased peptide recovery is not synonymous with restoration of a canonical wild-type repertoire. The latter group may include atypical ligands, peptides poorly modelled by current prediction algorithms, or co-purifying species and should therefore be interpreted cautiously.

The ERAP1^KO^ phenotype illustrates clearly the distinction between quantitative rescue and restoration of processing fidelity. ERAP1 normally removes N-terminal residues from peptide precursors and can either generate or destroy individual HLA-I ligands, with a strong influence on the abundance of optimally sized peptides [18,19,59]. Consistent with this role, ERAP1-deficient cells retained a broad length distribution enriched for 10–13mers and terminally extended ligands even after IFNγ treatment. IFNγ therefore increased the amount of material entering the antigen-presentation pathway but could not substitute for the missing trimming step. The persistence of the ERAP1-dependent signature is also consistent with pharmacological studies in which ERAP1 inhibition substantially remodels the immunopeptidome rather than simply reducing its size [60,61]. The selective reduction in HLA-A*02:01 surface expression in ERAP1^KO^ cells further suggests that the requirement for optimal ER trimming differs between the endogenous HAP1 HLA allotypes. This finding reinforces the view that ERAP1 perturbation should be considered in an HLA-specific context, particularly when ERAP1 inhibition is contemplated as a strategy to generate novel tumour-associated peptide repertoires.

The allele-selective effects observed following ERAP1 deletion further emphasise that the consequences of altered ER peptide trimming depend on the HLA molecules available for loading. Loss of ERAAP in mice has previously been associated with reduced MHC-I surface expression [62,63], whereas reduced ERAP1 expression in human monocytic cells increased the accumulation of aberrant HLA-B27 species [51] without producing equivalent changes in HLA-B18 or HLA-B51 [64]. Notably, HAP1 cells have previously been reported to lack ERAP2 expression based on RNA-sequencing data [25], indicating that the effects of ERAP1 deletion examined here were on a background without detectable endogenous ERAP2 expression. Together with the selective reduction in HLA-A*02:01 observed here, these findings indicate that dependence on ERAP1-mediated trimming varies substantially between HLA allotypes. Thus, the effect of ERAP1 deficiency should not be viewed simply as a global reduction in peptide maturation, but as an allotype-dependent redistribution of the peptide repertoire in which individual HLA molecules differ in their capacity to acquire stable ligands in the absence of optimal ER trimming.

The HLA-allotype binding-prediction analysis provides further evidence that disruption of peptide processing alters the contribution of individual HLA allotypes. In the untreated TAP-deficient immunopeptidomes, assignment of peptides to HLA-B*40:01 was markedly reduced, while a considerable fraction of the TAP2^KO^ repertoire was not predicted to bind any of the three endogenous HLA-I molecules using NetMHCpan 4.1 [50]. This poor representation of predicted HLA-B40 ligands demonstrates a strong dependence of this allotype on conventional TAP-mediated peptide supply, consistent with previous evidence that HLA-B allotypes differ substantially in their capacity to maintain antigen presentation during TAP deficiency [65]. The increased recovery of conventionally predicted ligands following IFNγ treatment therefore represents qualitative as well as quantitative rescue, although the persistence of substantial genotype-specific and poorly predicted peptide populations indicates that canonical allotype usage is not completely restored.

The TAP-deficient lines revealed a different form of plasticity. Both TAP1^KO^ and TAP2^KO^ cells displayed markedly reduced HLA-I surface abundance and peptide yield under basal conditions, as expected for disruption of the heterodimeric transporter that normally supplies the ER with cytosol-derived peptides. Nevertheless, IFNγ substantially increased HLA-I expression and immunopeptidome depth in both lines despite the fact that the deleted TAP subunit was not restored. This differs from tumour models in which IFNγ rescues antigen presentation principally by re-inducing low or suppressed TAP expression [66]. In the genetically TAP-deficient setting studied here, increased surface HLA-I must instead reflect a combination of increased HLA heavy-chain and B2M availability, altered peptide generation and greater use of TAP-independent peptide sources. TAP-independent HLA-I presentation is well established and can involve peptides generated from proteins entering the secretory pathway, including signal-peptide and intramembrane-processing routes [67]. The enrichment of peptides from the N-terminal regions of source proteins in TAP1^KO^ and TAP2^KO^ cells, together with shifts toward more hydrophobic peptide populations, is compatible with increased contribution from such ER-associated or membrane-proximal processing pathways, although the present data do not establish the origin of individual ligands.

An intriguing feature of the data is the different response of TAP1^KO^ and TAP2^KO^ cells to IFNγ. Following stimulation, TAP1^KO^ cells approached wild-type-like peptide-length and source-protein positional distributions more closely than TAP2^KO^ cells, whereas TAP2^KO^ retained a broader length profile, terminal extensions and stronger source-protein positional bias. Because canonical TAP function requires both TAP1 and TAP2, these differences should not be interpreted as evidence that one subunit can independently restore normal peptide transport. Rather, they suggest that loss of the two subunits generates distinct downstream states, potentially through differential compensatory changes in the remaining APM or altered access to non-canonical peptide sources. This interpretation is consistent with the broader PAKC analysis of Shapiro et al., in which individual APM knockouts generated distinct, pathway-consistent immunopeptidomic phenotypes even when the disrupted proteins participated in the same functional module [26].

The changes in peptide hydrophobicity and source-protein position further demonstrate that APM defects alter peptide quality as well as quantity. The persistence of higher hydrophobicity among subsets of knockout-specific ligands after IFNγ treatment indicates that inflammatory stimulation does not eliminate the physicochemical bias imposed by defective processing. Some of this effect may reflect altered access to membrane- or signal-sequence-derived peptides in the TAP-deficient lines, but changes in the relative contribution of HLA-A*02:01, HLA-B*40:01 and HLA-C*03:04 will also influence amino-acid composition because each allotype imposes distinct anchor requirements. Thus, peptide hydrophobicity should be interpreted as an emergent property of both processing and HLA selection rather than as a direct readout of a single enzymatic step. The same principle applies to the altered length distributions: peptide length reflects the combined effects of proteolysis, transport, ER trimming, peptide editing and the structural permissiveness of the individual HLA molecule.

The increased presentation of peptides derived from leukaemia-associated antigens (LAAs) after IFNγ treatment provides a clinically relevant illustration of this pathway plasticity. IFNγ broadened LAA-derived antigen presentation not only in HAP1^WT^ cells but also in cells lacking ERAP1, TAP1 or TAP2, indicating that inflammatory signalling can expose potentially targetable antigens even in the setting of defined antigen-processing defects. This observation is important for immunotherapy because the antigenic landscape encountered by T cells in an inflamed tumour may differ substantially from that measured under basal culture conditions. Previous work has similarly shown that IFNγ can diversify tumour immunopeptidomes and alter the relative presentation of candidate therapeutic targets [27,55,56].

Loss or dysfunction of HLA class I antigen-processing machinery is common across human cancers and provides an important route of tumour immune escape. The prevalence depends strongly on whether genomic alterations or loss of protein expression is considered. At the genomic level, somatic loss of heterozygosity (LOH) of the HLA-I locus occurs in approximately 17% of solid tumours, ranging from ~2 to 42% between tumour types and reaching ~30% in squamous carcinomas; in NSCLC, HLA-I LOH has been reported in approximately 40% of tumours in some cohorts [68,69]. Somatic HLA-I mutations are less frequent, occurring in approximately 3.3% of 7930 TCGA tumours, but are enriched in head and neck, lung squamous, gastric and colorectal cancers [70]. Alterations affecting other APM genes are similarly heterogeneous: analysis of head and neck cancers identified genomic alterations involving HLA-I and APM genes in approximately 20% of cases, with TAP1 and TAP2 altered in ~3–4% of individuals [71]. Somatic B2M mutations are relatively uncommon in unselected tumours, occurring in approximately 1.8% of cancers in a pan-cancer TCGA analysis and in ~1–5% of several unselected tumour cohorts, but are markedly enriched in hypermutated and immunogenic settings. For example, B2M mutations were detected in 24.2% of MSI-H colorectal cancers compared with only 0.9% of microsatellite-stable colorectal cancers [72,73]. Importantly, defective protein expression is substantially more frequent than structural mutation, reflecting transcriptional, epigenetic and post-transcriptional regulation. Across historical tumour series, reduced or absent TAP1/TAP2 expression has been reported in ≥40% of many cancer types, although frequencies are lower in breast and ovarian cancers (<30%), while abnormalities in HLA-I heavy-chain expression range from ~36% in renal cancer to nearly 88% in thyroid cancer [74]. Thus, complete genetic disruption of individual APM components is relatively uncommon, whereas partial loss, downregulation or allele-specific disruption of the antigen-presentation pathway is widespread, providing a strong biological rationale for understanding how inflammatory signals such as IFNγ compensate for individual APM lesions and reshape the residual immunopeptidome.

## 5. Conclusions

This study demonstrates that inflammatory signalling can substantially compensate for defined defects in the HLA-I antigen-processing machinery, but that increased HLA-I surface expression and peptide yield should not be equated with restoration of a wild-type immunopeptidome. IFNγ strongly induced the residual APM in HAP1 cells, expanded HLA-I ligand recovery and increased presentation of LAA-derived peptides across ERAP1-, TAP1- and TAP2-deficient backgrounds. Nevertheless, each genetic lesion retained a characteristic processing signature. ERAP1 deficiency preserved an extended-peptide phenotype despite IFNγ, while TAP2 deficiency remained associated with pronounced alterations in peptide length, terminal processing and source-protein position. TAP1-deficient cells showed a greater degree of convergence toward wild-type features, highlighting unexpected heterogeneity in the capacity of inflammatory signalling to compensate for defects within the same antigen-transport module.

Collectively, the findings support a model in which IFNγ provides quantitative and partial qualitative “repair” of antigen presentation by amplifying intact pathway components and increasing access to alternative peptide sources, rather than by bypassing the molecular requirement for the deleted APM component. This distinction has direct implications for tumour immune escape and antigen-targeted therapy: APM-deficient cancer cells may remain visible to T cells under inflammatory conditions, but the peptides they present can differ substantially from those displayed by antigen-processing-competent cells. Defining these defect- and inflammation-specific immunopeptidomes may therefore reveal both mechanisms of immune evasion and antigenic vulnerabilities that are not apparent from analysis of the untreated cellular state.

## Data Availability

The mass spectrometry proteomics and immunopeptidomics data generated in this study has been deposited with the ProteomeXchange Consortium via the PRIDE partner repository [75]. The corresponding ProteomeXchange/PRIDE dataset accession number(s) contain the immunopeptidomics (PXD085470) and proteomics (PXD085506) data.

## Supplementary Materials

The following supporting information can be downloaded at https://www.mdpi.com/article/10.3390/cells15201914/s1: Figure S1: IFNγ treatment significantly upregulates cell surface HLA-I levels in HAP1 cells; Figure S2: Knockout of APM components significantly increases the presentation of hydrophobic peptides; Table S1: Proteomics data; Table S2: Immunopeptidomics data; Table S3: HC-10 LC-MS spectral count data.

## Abbreviations

The following abbreviations are used in this manuscript:

ACN	Acetonitrile
APM	Antigen Presentation and Processing Machinery
CML	Chronic Myelogenous Leukaemia
cRAP	Common Repository of Adventitious Proteins
DDA	Data-Dependent Acquisition
DIA	Data-Independent Acquisition
DMSO	Dimethyl-Sulphoxide
ER	Endoplasmic Reticulum
ERAP	Endoplasmic Reticulum Aminopeptidase
FA	Formic Acid
FDR	False Discovery Rate
gMFI	Geometric Mean Fluorescence Intensity
GSEA	Gene Set Enrichment Analysis
HC	Heavy Chain
HLA	Human Leukocyte Antigen
HLA-I	HLA Class I
IFNγ	Interferon γ
iRT	Indexed Retention Time
LC-MS/MS	Liquid Chromatography–Tandem Mass Spectrometry
PAKC	Panel Of HLA Class I Antigen Presentation Machinery Knockout Cells
PBS	Phosphate-Buffered Saline
PFA	Paraformaldehyde
PLC	Peptide-Loading Complex
PTM	Post-Translational Modification
PΩ	C-Terminal Anchor Residue
RP-HPLC	Reversed-Phase High-Performance Liquid Chromatography
RT	Room Temperature
TAA	Tumour-Associated Antigen
TAP	Transporter-Associated with Antigen Processing
TFA	Trifluoroacetic Acid
β2m	Beta-2-Microglobulin
