# Supplementary material for: Cytokine-Mediated Plasticity in the Molecular Requirements for Peptide-Loading Complex Formation and Antigen Presentation
Source: Cells. 2026 Oct 21;15(20):1914. doi: 10.3390/cells15201914 (PMC13703434; doi:10.3390/cells15201914)
Supplement: Supplementary file 1 [file cells-15-01914-s001.zip › Figure S1.pdf]

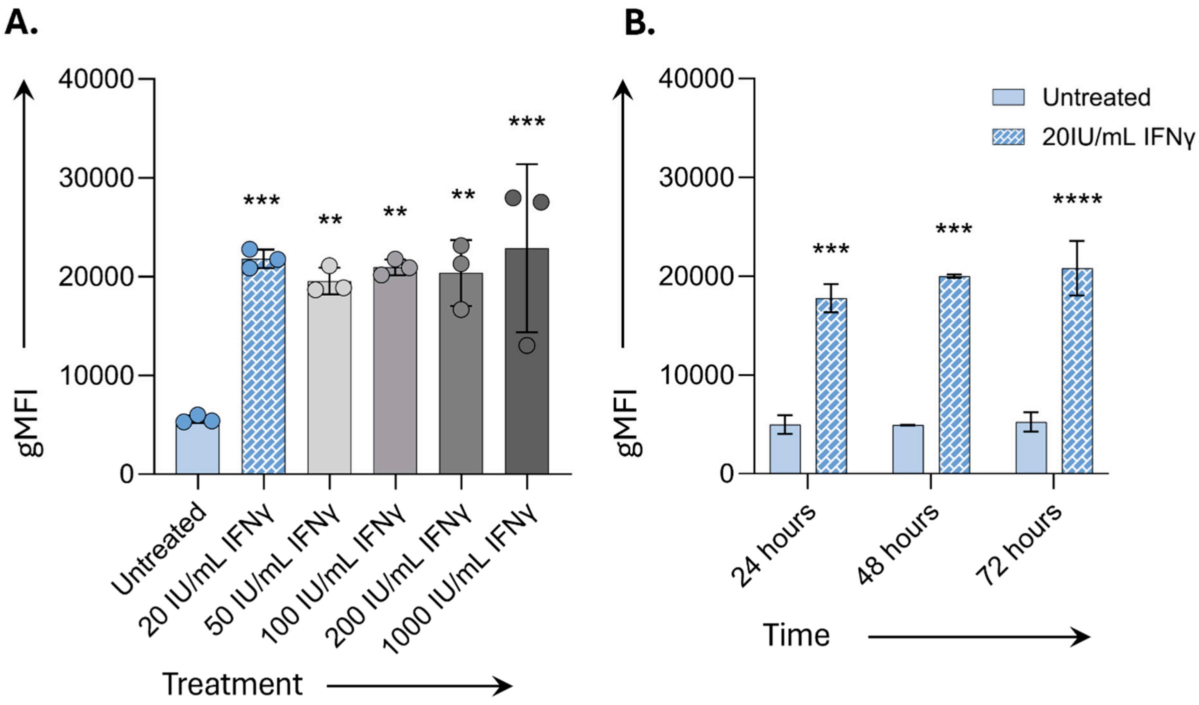

Supplemental Figure S1: IFNγ treatment significantly upregulates cell surface HLA-I levels in HAP1 cells. Flow cytometry was used to quantify cell surface HLA-I expression of wildtype Hap1 ($HAP1^{WT}$) cells following IFNγ stimulation using W6/32 (pan HLA-I antibody) as primary antibody. **(A)** $HAP1^{WT}$ cells were treated for 24 hours at different concentrations of IFNγ (20 IU/mL, 50 IU/mL, 100 IU/mL, 200 IU/mL, and 1000 IU/mL) represented as (mean ± SD), n=3. **(B)** Time course assay of $HAP1^{WT}$ cells treated at 20 IU/mL IFNγ, at 24, 48, and 72 hours represented as (mean ± SD).
