# Supplementary material for: Cytokine-Mediated Plasticity in the Molecular Requirements for Peptide-Loading Complex Formation and Antigen Presentation
Source: Cells. 2026 Oct 21;15(20):1914. doi: 10.3390/cells15201914 (PMC13703434; doi:10.3390/cells15201914)
Supplement: Supplementary file 1 [file cells-15-01914-s001.zip › Figure S2.pdf]

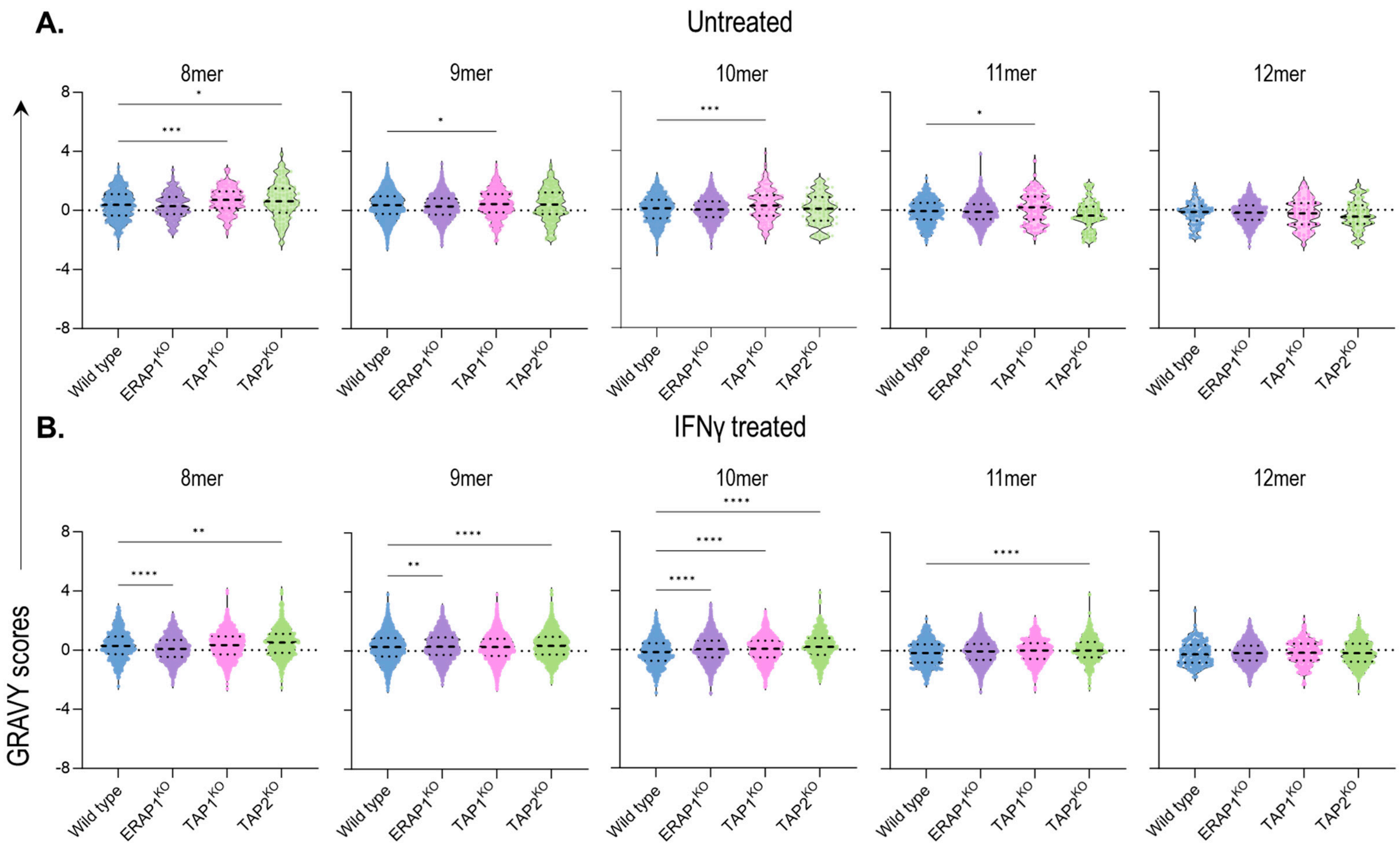

Figure S2: Knockout of APM components significantly increases the presentation of hydrophobic peptides. Peptide hydrophobicity scores were calculated using Kyte and Doolittle scale with hydrophobic amino acids scoring on the positive Y-axis and hydrophilic amino acids scoring on the negative Y-axis. Average hydrophobicity scores of 8-12mer peptides unique to each cell line presented in (**A**) untreated cells and (**B**) IFNγ treated cells. Violin plots represent hydrophobicity scores, with dashed line representing the median, and dotted lines representing the quartiles. Significance determined using an Ordinary one-way ANOVA followed by Tukey's multiple comparisons test, with a single pooled variance. (**** $p \leq 0.0001$, *** $p \leq 0.001$, ** $p \leq 0.01$, * $p \leq 0.05$)
